# Analyzing the adoption of AIGC tools in fashion design: An S-O-R framework integrating task–technology fit

**DOI:** 10.1371/journal.pone.0335522

**Published:** 2025-10-27

**Authors:** Mengyun Yang, Jiabing Jin

**Affiliations:** 1 Faculty of Creative Arts and Design, UCSI University, Kuala Lumpur, Malaysia; 2 School of Fashion Design, Jiangxi Institute of Fashion Technology, Nanchang, China; 3 School of Textile Science and Engineering, Jiangnan University, Wuxi, China; Al-Ahliyya Amman University, JORDAN

## Abstract

AI-Generated Content (AIGC) tools have rapidly emerged in the field of apparel design in recent years, but how designers adopt these tools and the psychological mechanisms behind them are unclear. This study constructs a model based on the stimulus-organism-response (S-O-R) theory, aiming to reveal how external stimulus variables (perceived content quality (PCQ), personalized fit (PF), industry pressure (IP), perceived technological risk (PTR)) are adopted through psychological state variables (self-efficacy (SE), innovativeness (INN), and task technology fit (TTF)) influence apparel designers’ AIGC adoption intentions. Based on the questionnaire data of 267 Chinese fashion designers, partial least squares structural equation modeling (PLS-SEM) was used for empirical analysis. The results showed that: PCQ and PF significantly enhanced SE and TTF, while PTR significantly inhibited the above psychological mechanisms; SE, INN, and TTF positively influenced adoption intention, with TTF having the most significant effect, and IP did not show a significant effect. The findings not only validate the applicability of the S-O-R theory in creative technology adoption, but also emphasize the key role of TTF matching and psychological-cognitive factors in promoting the application of AIGC tools, providing theoretical support and practical insights for subsequent tool optimization and user guidance.

## 1. Introduction

In recent years, emerging technologies represented by AI-Generated Content (AIGC) have rapidly emerged in the creative design field. Generative AI technologies (e.g., GAN and diffusion models) have revolutionized the fashion industry by supporting the rapid creation of innovative designs [1]. AIGC provides designers with new inspirations and tools by generating creative content, such as images, texts, and videos, through deep learning models (e.g., GPT, DALL-E, Midjourney, etc.) [2]. Nevertheless, designers are also cautious about AIGC. On the one hand, AIGC helps to quickly generate design solutions and provide creative ideas; on the other hand, some experts are concerned that it may lower the design quality standard and cause plagiarism and copyright issues [3,4]. In addition, there is still little literature focusing on how the community of fashion designers, who rely on original expressions, perceive and accept AIGC as an emerging tool in their actual creations.

Previous studies on the adoption of AI have primarily examined the industrial or service sectors and have primarily employed the Unified Technology Acceptance and Use Model (UTAUT) or the Technology Acceptance Model (TAM) to explain their findings [5,6]. However, these standard models are inadequate for describing the adoption of AIGC, a creative and non-deterministic technology, because they fail to capture the deep psychological and emotional characteristics of creative professionals [7]. To address this limitation, recent research has used the Stimulus-Organism-Response (S-O-R) framework to provide a more comprehensive view. Wang and Chen used variables from TAM and TPB into the S-O-R model to investigate creative designers’ use of AIGC [4]. Similarly, Huang et al. demonstrated how contextual factors promote adoption via self-efficacy [8]. While these studies are informative, they do not adequately address the basic issues that AIGC raises to current technology adoption theories.

Although the S-O-R framework has shown strong explanatory flexibility in traditional technology adoption research [9], two theoretical limitations emerge when it is applied to generative AI in creative contexts. These limitations form the core entry point of this study. First, there is insufficient attention to the unique nature of creative tasks. Most S-O-R studies stem from relatively structured and goal-oriented settings, such as e-commerce and online services [10]. By contrast, fashion design tasks are inherently unstructured, exploratory, and strongly tied to individual style expression [11,12]. Existing models overlook the central mediating role of task–technology fit (TTF) in this context [13]. Yet TTF is crucial for determining whether AIGC evolves from an interesting novelty into a genuinely useful production tool. Second, current research has not systematically integrated key psychological constructs specific to creative professionals. The generative nature of AIGC makes it resemble a creative partner rather than a passive tool. This calls for adoption models to include psychological variables such as innovativeness [14] and self-efficacy [15]. These reflect an individual’s tendency to explore the unknown and their belief in handling complex AI tools, thereby capturing the decision-making process of designers as “creative agents.” Existing studies have paid limited attention to this dimension.

To address these limitations, this study develops a modified S-O-R model to fill the identified gaps. (1) It limits the study’s focus to the usage of generative AI in fashion design, looking into how the S-O-R framework functions in this context. (2) It highlights TTF in the model, claiming that TTF is the basic connection between the AIGC stimuli and designers’ adoption responses. (3) It systematically combines important psychological constructs such as self-efficacy [15] and innovativeness [14] into a variable system that is more closely related to the cognition of creative fields. By focusing on these three characteristics, this study goes beyond a broad discussion of technology adoption to give a more relevant theoretical lens for understanding how AIGC might be integrated into creative professional work.

This paper’s structure is set up as follows: The conceptual model and hypothetical course of this study are presented in Section 2, which is based on an analysis of the theoretical underpinnings of S-O-R and associated literature. The study’s methodology, including the questionnaire design, sample recruitment standards, data collecting, and scale measurement, is covered in Section 3. Section 4 presents the results of the empirical analyses, including the assessment of the measurement model, the structural model, and the predictive validity. Section 5 combines the path analysis and IPMA results to provide an in-depth discussion of the theoretical contribution and practical significance of the study’s findings, as well as to point out the limitations and future research directions of this study.

## 2. Literature review

### 2.1. Application of S-O-R theory to technology acceptance

Mehrabian and Russell proposed S-O-R theory to describe how individuals’ psychological states and behavioral response mechanisms change in response to specific environmental stimuli [16]. The model suggests that the stimulus (S) is an external environmental factor that triggers a change in the internal cognitive or affective state of the organism (O), resulting in a corresponding response (R). In recent years, SOR models are often applied to consumer behavior, e-commerce, and acceptance of virtual reality and artificial intelligence technologies [17–19].

From a complete perspective, the S-O-R model shows the following three advantages in technology adoption research: 1. Clear structure: it can effectively delineate the three stages of technology acceptance path: “external environmental stimulus - psychological perception change - behavioral response”; 2. Flexible variables: it can integrate different theoretical variables (e.g., PU/PEOU in TAM, motivation variable in SDT); 3. Suitable for contextualized research, such as fashion design, adoption of technology for the elderly, AI customer service systems, and other fields that emphasize “psychological regulation” and “perceptual factors”.

However, current S-O-R-based research on technology adoption have two major shortcomings. First, most studies have concentrated on structured and goal-oriented contexts, such as e-commerce, online services, or smartphone use among older persons [13,14,16]. The work qualities in these contexts are fundamentally different from creative activities in fashion design, which are unstructured, exploratory, and heavily reliant on personal expression. Applying models from structured settings, such as Huang’s study on smartphone adoption among older adults [8], to AIGC-assisted creative scenarios risks overlooking the critical role of task-technology fit (TTF) and the complex psychological motivations underlying creative work. Second, present research pays little attention to the internal psychological characteristics within the “organism,” particularly critical variables specific to creative professionals, such as innovativeness and self-efficacy in creative expression. Although Vafaei-Zadeh et al. used TTF, they focused on functional fit and emotional trust in AI customer service systems [20]. In contrast, AIGC functions as a creative collaborator rather than a passive instrument, including various psychological mechanisms such as the desire for autonomy and originality. Taken together, these gaps emphasize the need to adapt and reconstruct the S-O-R model’s variables and pathways in order to effectively reflect technology adoption in creative settings.

### 2.2. Integrating SDT and DOI into the S-O-R framework

The S-O-R model outlines the macro pathway of “stimulus–organism–response” for this study. To better explain the psychological mechanisms behind fashion designers’ adoption of AIGC, and to provide stronger theoretical grounding for the research variables, we further examine the connections between our conceptual model and two classical theories: Self-Determination Theory (SDT) [21] and the Diffusion of Innovations (DOI) theory [22]. Together, these theories offer powerful lenses for interpreting the roles of the variables in our framework.

Most prior studies still rely on the traditional perceived usefulness and ease of use paradigm [23] or focus only on external environmental pressures [13]. They fall short in revealing how AIGC adoption is driven by the fulfillment of creative professionals’ intrinsic psychological needs, such as autonomy and competence. To bridge this gap, our study operationalizes the SDT construct of autonomy as personalization fit (PF) and the construct of competence as perceived content quality (PCQ). At the same time, DOI highlights that adoption depends on both the attributes of the innovation (e.g., compatibility, relative advantage) and the characteristics of the adopters (e.g., innovativeness) [22]. When AIGC is viewed as an innovation, our variables align closely with DOI: TTF reflects the compatibility between AIGC and design tasks while also signaling its relative advantage; innovativeness (INN) represents a core individual trait that distinguishes early from late adopters; and perceived technological risk (PTR) corresponds to the uncertainty inherent in innovation. In this way, the S-O-R framework provides the macro structure, while SDT and DOI enrich it with micro-level theoretical insights. Together, they allow for a more comprehensive understanding of the psychological mechanisms influencing fashion designers’ adoption of AIGC.

### 2.3. Relationship between stimulus and organism

#### 2.3.1. Impact path of personalization fit.

Personalized fit (PF) refers to a user’s perception of an information system’s ability to provide tailored functionality and content to meet his or her unique needs based on his or her individual preferences, usage habits, and behavioral characteristics [24]. This concept has been widely used in human-computer interaction and recommender systems research, and is recognized as a key technical attribute for enhancing users’ perceived value, satisfaction and willingness to use [25]. As AIGC continues to expand its applications in the creative design field, personalization capabilities are becoming an important mechanism for enhancing user expression. For fashion designers who rely on style presentation and creative freedom, whether the system has the ability to highly match their personalized needs has become a key factor affecting their experience and creative motivation [26,27].

According to SE theory, personalization features help to increase users’ sense of control and confidence in the operation of the system, thereby enhancing their SE in specific tasks [28]. For example, in a study by Komiak and Benbasat, it was found that the level of personalization of the system significantly increased the user’s trust in the system and boosted his or her confidence in taking control of the task [25]. In addition, Tam and Ho noted that personalized systems are effective in reducing cognitive load and increasing users’ expectations of efficacy during information processing [29]. Therefore, this study proposes the following hypothesis:

H1: Personalized fit positively affects fashion designers’ self-efficacy.

In addition, PF also contributes to TTF, which is the user’s overall judgment of whether the system functionality fits his or her task needs. Goodhue and Thompson point out that when the system functionality is highly matched to the user’s task, the user is more likely to perceive the value of the technology’s assistance, which in turn increases the motivation to use it [30]. In the scenario of AIGC-assisted design, if the system can provide differentiated outputs and prompts based on designers’ creative styles and design paths, it is more likely to enhance their perception of the system’s “suitability” and consider the tool as a technological means to effectively support their creative tasks [31]. In the process, personalization not only enhances efficiency of use, but also promotes recognition of the fit between task completion and system capabilities. Therefore, this study proposes the following hypothesis:

H2: Personalized fit positively affects fashion designers’ task-technology fit.

#### 2.3.2. Impact path of perceived content quality.

PCQ refers to the user’s overall evaluation of the AI system-generated content in terms of accuracy, completeness, aesthetics and innovativeness [32,33]. In the application of AIGC to creative design contexts, content quality not only determines the designer’s trust in the system, but also affects his subjective judgment of task supportability. For fashion designers, high-quality content is not only the basis for creative expression, but also largely affects their perception of the system’s capabilities, their own performance and the degree of task completion [34].

Bandura states that SE is an individual’s belief in his or her ability to accomplish a specific task [28]. When designers perceive that the content generated by the AIGC system is of high quality, they are more likely to believe that they can effectively utilize the system to accomplish their design tasks, thus increasing their SE. McGill & Klobas’ empirical study also noted that clarity and structural integrity of content in an information system significantly increased users’ confidence in completing tasks, a finding that applies to the use of AIGC tools in design tasks [31]. Therefore, this study proposes the following hypothesis:

H3: Perceived content quality positively influences fashion designers’ self-efficacy.

Quality content can stimulate users’ desire for creative expression and enhance their motivation to create. Runco and Jaeger suggested that innovativeness is significantly influenced by contextual factors, and that individuals are more likely to generate novel ideas when external stimuli are original and expressive [35]. Guo et al.’s study noted that AI assisted design system enhanced designers’ creative expression and design satisfaction by providing high-quality personalized content [26]. Therefore, this study proposes the following hypothesis:

H4: Perceived content quality positively influences fashion designers’ innovativeness.

TTF refers to the degree to which the functionality of a technology system matches the user’s task requirements. In the classic TTF theory that the ability of a system to provide the “high quality of information content” required by the user’s task is an important factor in determining the level of TTF [30]. McGill and Klobas pointed out that the higher the quality of the system’s content, the easier it is for the user to perceive its ability to support the task, which enhances the agreement on the system’s fit with the task [31]. Therefore, this study proposes the following hypothesis:

H5: Perceived content quality positively influences fashion designers’ task–technology fit.

#### 2.3.3. Impact path of industry pressure.

Industry pressure (IP) refers to the external driving force felt by an individual or an organization in the face of market changes, industry trends, technological changes, competitor actions, and customer expectations [36]. In the field of creative design, especially in the face of the rapid rise of new technologies such as AIGC, fashion designers are faced not only with the challenge of technological transformation, but also with multiple pressures from peer adoption, brand competitiveness, and design delivery efficiency. This pressure is sometimes not only an external threat, but also a “challenging stimulus” that stimulates designers’ innovativeness and technology-adaptive behavior.

Findings on the effect of industry pressure on creative behavior remain inconsistent. While Zhou and George suggested that pressure may spur creativity [37], other study found that time pressure or competitive pressure can, in some cases, inhibit exploratory innovation [38]. In the emerging context of AIGC, it is still unclear whether industry pressure works as a “challenge stressor” that encourages tool exploration, or as a “hindrance stressor” that leads to conservative decision-making. Moreover, empirical proof is particularly lacking for fashion designers as a specific group. By introducing this variable, the present study aims to test this uncertain relationship and clarify the real role of industry pressure in creative technology adoption. Therefore, the following theory is proposed:

H6: Industry pressure positively influences fashion designers’ innovativeness.

In addition, IP may also affect designers’ perceptions of the fit between the AIGC system and their daily design tasks. According to the study presented by Teo et al., environmental factors such as market dynamics, regulatory changes and industry standards can significantly influence an individual’s evaluation of the adaptability of a new technology [36]. Therefore, this study proposes the following hypothesis:

H7: Industry pressure positively influences fashion designers’ task–technology fit.

#### 2.3.4. Impact path of perceived technology risk.

PTR refers to the subjective concerns that users have about possible negative consequences, such as privacy breaches, output errors, lack of controllability, or uncertainty, when using a technological system [39]. In the context of AIGC applied to creative design, fashion designers may worry about inaccurate generation results, uncontrollable content, infringement of originality, or design styles that deviate from their intentions. These perceived risks not only inhibit their willingness to adopt the technology, but also significantly affect their self-assessment of competence and judgment of system task fit. First, PTR may reduce designers’ self-efficacy. According to Bandura’s social cognitive theory, self-efficacy is derived from task success experiences and expectations of outcome control [28]. If designers perceive the AIGC system as having a high level of uncertainty or unpredictable risk of failure, they may doubt their ability to effectively navigate the technology, thereby undermining their confidence in their own design abilities. Relevant research has shown that uncertainty, system complexity, and potential errors in technology use significantly reduce users’ assessments of their ability to accomplish tasks [15]. In the context of AI system use scenarios, users’ self-efficacy significantly decreases when they perceive system outcomes as unexplainable or unpredictable [40].

H8: Perceived technology risk negatively affects fashion designers’ self-efficacy.

Second, the perception of technology risk also affects the judgment of TTF. TTF is based on users’ trust in the reliability and usefulness of the technology [30]. If designers perceive the AIGC system to have problems such as stylistic drift, logical errors, or content uncertainty, they may believe that the technology is unable to meet the high demands of their daily design tasks, thus reducing their agreement on the degree of system-task fit. Similar to findings in the field of online banking, Martins et al. demonstrated that perceived risk significantly undermines user trust and adoption intention toward technological systems. Extending this logic to creative design contexts, designers may also lower their evaluation of AIGC tools’ applicability when risks such as content quality or copyright issues are perceived [41]. Therefore, this study proposes the following hypothesis:

H9: Perceived technology risk negatively influences fashion designers’ task technology fit.

### 2.3. The influence of organism on response

#### 2.3.1. Effect of self-efficacy on adoption intention.

SE is a person’s belief in his or her ability to accomplish a task and is one of the important cognitive variables in determining whether to adopt a new technology. The stronger the SE, the more likely an individual is to persist and adopt proactive behaviors in the face of challenges [28]. In the context of this study, SE refers to a user’s self-assessment of his or her confidence and ability to use assistive design technologies. When AIGC tools are matched to a designer’s specialized skills and knowledge, the designer finds it easier to learn and use the tools effectively. This congruence increases designers’ confidence in their ability to use the new technology, which in turn increases their self-efficacy. A strong positive correlation between SE levels and acceptance of AI has been confirmed in one study [42]. Higher levels of user self-efficacy in the AIGC design process can instill confidence in the operation of AIGC-assisted technology, leading to a more positive affective and attitudinal approach to learning and interacting in AIGC-assisted design and a greater willingness to invest time and effort to achieve goals. Based on this, the study proposes the following hypothesis:

H10: Self-efficacy positively influences fashion designers’ intention to adopt AIGC.

#### 2.3.2. The impact of innovativeness on adoption intention.

INN refers to an person’s propensity to actively try and embrace new technologies or tools when faced with them, and is a key individual characteristic variable in technology adoption research [43]. Unlike general creativity, innovativeness emphasizes the behavioral willingness and tendency to adopt and experiment with new technologies, especially in the context of rapid digitalization, highly innovative practitioners are more inclined to take the lead in adopting emerging tools that can enhance efficiency and improve expressiveness.

According to Rogers’s study, innovativeness is a key indicator of whether an individual is an “early adopter”. Such individuals have a high tolerance for uncertainty and tend to explore new technologies proactively to gain a competitive advantage, especially when the tools offer greater freedom of expression and design flexibility [44]. Yoo et al. (2010) further point out that in technology-driven creative innovation, the positive response of individuals to digital tools is the basis for the continuous evolution of organizational and individual innovation behaviors.

Goldsmith and Hofacker proposed that innovativeness is one of the most stable psychological variables explaining technology adoption intentions, especially in creative endeavors that emphasize individual expression, rapid iteration, and a high degree of individual stylization [45]. As a result, the more innovative the apparel designer, the more likely it is that he or she will demonstrate a more positive intent to adopt AIGC out of a quest for technological sensitivity and creative breakthroughs. Therefore, this study proposes the following hypothesis:

H11: Innovativeness positively influences fashion designers’ intention to adopt AIGC.

#### 2.3.3. Impact of task technology fit on adoption intentions.

TTF refers to whether the functionality of a technological system can effectively meet the needs of users to perform a specific task, emphasizing that when the technology is a high fit with the user’s work task, the individual will be more likely to use the technology and exhibit higher levels of behavioral intent and performance [30]. In the context of the gradual integration of AIGC into the creative design process, fashion designers who perceive that AIGC tools can efficiently meet their design needs in terms of idea generation, image reconstruction, and text-image collaboration are more likely to develop a positive intention to adopt them.

In recent years, TTF has been widely used in acceptance studies of AI systems, information recommendation platforms, and smart tools. McGill and Klobas found in a study of learning management systems that the degree of content-functionality fit had a significant impact on users’ technology use effectiveness and satisfaction [31]. This finding also applies to AIGC system usage scenarios in creative tasks. Butt et al. in their study of the effect of task technology fit on digital platform adoption, found that when users perceived a high degree of fit between system functionality and their task needs, not only did they increase their frequency of use of the system, but they also significantly enhanced their positive perceptions of platform efficacy [46]. Although this study was conducted in the context of an e-learning platform, the results are also applicable to task-driven creative design processes. For fashion designers, if the AIGC system demonstrates a high degree of fit in key areas such as content generation, style control and design collaboration, designers are more likely to recognize its professional value and actively adopt the tool to assist in their creative endeavors. In summary, combining TTF theory with existing research results, this paper proposes the following hypotheses:

H12: Task technology fit positively influences fashion designers’ intention to adopt AIGC.

**Fig 1:**
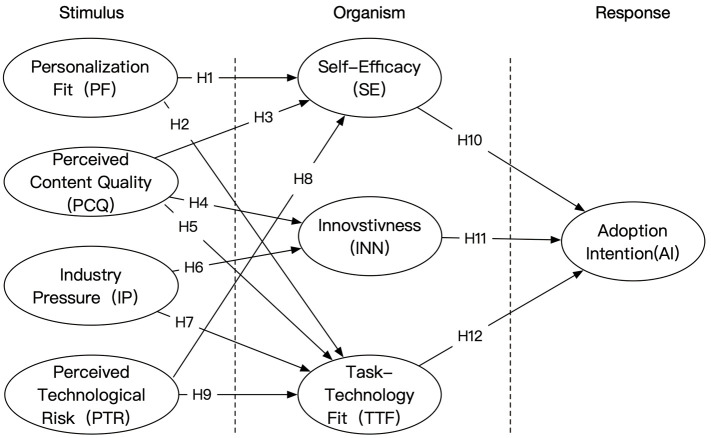
Research model.

This study builds an overall structural framework based on the S-O-R theory, which integrates technical characteristics (e.g., PF, PCQ), environmental pressures (e.g., IP, PTR) and individual psychological factors (e.g., INN, SE, and TTF) to systematically explore the mechanisms of their influences on the intention of fashion designers to adopt the AIGC technology. Based on the theoretical derivation and literature review, a total of 12 hypothesized paths were proposed, and the conceptual model of the study was constructed accordingly (see Fig 1).

## 3. Methodology

### 3.1. Questionnaire design and pilot testing

This study employed a questionnaire survey to collect data. The design of the questionnaire was based on a review of the literature and expert consultation to ensure strong content validity of the constructs. All measurement items were assessed on a 7-point Likert scale (1 = “strongly disagree,” 7 = “strongly agree”). The full list of items and their sources is provided in S1 Table. Before formal distribution, the questionnaire was refined through two pilot tests. In the first stage, three scholars with expertise in artificial intelligence and fashion design reviewed the items for clarity, relevance, and contextual appropriateness. In the second stage, 20 fashion designers with AIGC experience completed a trial survey. The reliability of each construct was evaluated using Cronbach’s *α*, with all values exceeding 0.70. Based on their feedback, several items were revised to reduce social desirability bias and improve comprehensibility. The strategies included: (1) rephrasing items to use neutral language and avoid leading questions; (2) emphasizing in the survey instructions that there were no right or wrong answers and that anonymity was guaranteed; and (3) ensuring the questions focused on practical behaviors and experiences rather than personal ethics or competence.

### 3.2. Sample and data collection

This study adopted purposive sampling. The questionnaire was distributed through online communities, professional associations, and social media groups related to fashion design (e.g., WeChat groups and QQ design forums). Participants were required to have used at least one AIGC tool (such as Midjourney, DALL·E, Stable Diffusion, or Blibli) for fashion design tasks within the past six months. To ensure this criterion, a screening question was included at the beginning of the survey (“Have you used an AIGC tool for fashion design in the last six months?”). Only those who answered “yes” were able to proceed.

Before starting the survey, participants were presented with a clear informed consent statement on the first page of the questionnaire. They were required to check a consent box to indicate their agreement to participate voluntarily and anonymously. Only those who provided consent were allowed to proceed with the questionnaire, while those who declined were automatically exited from the survey. The study was conducted in accordance with the Declaration of Helsinki. Ethical review and approval were waived for this study because it involved an anonymous questionnaire, did not include personal sensitive information, posed no harm to individuals, and was not associated with commercial interests. The legal basis for waiving ethical review and approval is Article 32 of the Ethical Review Measures for Life Sciences and Medical Research Involving Humans, promulgated by the National Health Commission of China (https://www.gov.cn/zhengce/zhengceku/2023-02/28/content_5743658.htm, published on 18 February 2023).

The questionnaire was distributed through the Wenjuanxing platform, and 335 responses were collected. After removing incomplete answers, patterned responses, and those failing the attention-check items, 267 valid samples were retained. The main demographic characteristics of the sample are shown in Table 1. Although the participants were mainly from mainland China, they represented a diverse range of ages, educational backgrounds, and professional types, which provides a reasonable reflection of the current structure of active designers in the industry.

**Table 1:** Demographic profile (n = 267).

Demographic	Frequency	Percentage (%)
Gender		
Male	72	26.97%
Female	195	73.03%
Age		
18-25	102	38.20%
25-35	108	40.45%
36-45	52	19.48%
46+	5	1.87%
Educational background		
High School or Below	35	13.11%
Bachelor’s Degree	205	76.78%
Graduate Degree or Higher	27	10.11%
Position		
Independent Designer	34	12.73%
Designer in Fashion Enterprises	128	47.94%
Fashion Academia/Research Staff	105	39.33%

## 4. Results

### 4.1. Common method bias (CMB)

In order to mitigate the possible impact of CMB on the results of the study, this study combined both procedural control and statistical testing, referring to the recommendations of Podsakoff et al. for the treatment [47]. In terms of procedural design, experts in the field of artificial intelligence and fashion design were invited to participate in the questionnaire design phase to ensure the content validity and linguistic clarity of the measurement items. All items were expressed in a clear and concise manner to avoid ambiguity, and the measures with different conceptualizations were randomized to reduce the responding tendency of the subjects. In addition, at the beginning of the questionnaire, respondents were informed that their responses were for academic purposes only, were completely anonymous, and that there was no right or wrong answer, so as to reduce their evaluation apprehension. In terms of statistical tests, this study used Harman’s Single Factor Test to test for common method bias. The results showed that the total variance explained by the first factor in the unrotated principal component analysis was 17.216%, which is well below the conservative threshold of 40%, indicating that common-method bias does not pose a serious threat. In addition, the results of the correlation matrix between the variables showed that the highest correlation coefficients between the constructs were below 0.90, further indicating that this study has good discriminant validity.

### 4.2. Partial least squares structural equation modeling (PLS-SEM)

Following the recommendations of Hair et al., this study used partial least squares structural equation modeling (PLS-SEM) as the primary data analysis method [48]. First, PLS-SEM is suitable for prediction-oriented research and theory development-oriented modeling, which fits the research purpose of this study to explore designers’ intention to adopt AIGC. Second, PLS-SEM is suitable for handling non-normally distributed data, which is widely used in social science research contexts. Third, considering the multiple mediating paths included in this study’s model, PLS-SEM is more suitable for estimating the path coefficients of latent variables under a complex model structure and does not require strict sample size requirements.

#### 4.2.1. Measurement modeling.

At the measurement modeling stage, this study first assessed the reliability and validity of each latent variable. The combined reliability (CR) of all constructs was above 0.70 according to Hair et al.’s criteria, indicating good internal consistency [48]. The average variance extracted (AVE) also all exceeded the recommended threshold of 0.50, indicating that the latent variables had sufficient convergent validity [49]. Also in this study, Dijkstra-Henseler’s rho_A, which is considered to be more applicable to PLS-SEM modeling than Cronbach’s α, was calculated, and all of its values were greater than 0.70, further validating the reliability of the constructs [50]. In summary, the internal consistency and convergent validity of the measurement models were met. Subsequently, the discriminant validity between the constructs was assessed using the heterotrait-monotrait ratio (HTMT). The HTMT values were all below 0.90 [51], indicating that the constructs were well differentiated from each other and the measurement model possessed satisfactory discriminant validity. The related detailed statistics are shown in Table 2 and Table 3.

**Table 2:** Construct reliability and convergent validity.

Latent variables	Items	Loadings	CR	rho_A	AVE
Personalized Fit (PF)	PF1	0.866	0.918	0.911	0.691
	PF2	0.857			
	PF3	0.818			
	PF4	0.792			
	PF5	0.821			
Perceived Content Quality (PCQ)	PCQ1	0.866	0.890	0.906	0.602
	PCQ2	0.805			
	PCQ3	0.715			
	PCQ4	0.787			
	PCQ5	0.755			
Industry Pressure (IP)	IP1	0.833	0.826	0.893	0.548
	IP2	0.878			
	IP3	0.849			
	IP4	0.744			
Perceived Technological Risk (PTR)	PTR1	0.867	0.917	0.965	0.690
	PTR2	0.808			
	PTR3	0.806			
	PTR4	0.835			
	PTR5	0.835			
Self-Efficacy (SE)	SE1	0.830	0.921	0.904	0.700
	SE2	0.865			
	SE3	0.818			
	SE4	0.820			
	SE5	0.849			
Innovativeness (INN)	INN1	0.864	0.914	0.905	0.680
	INN2	0.849			
	INN3	0.818			
	INN4	0.770			
	INN5	0.818			
Task-Technology Fit (TTF)	TTF1	0.883	0.843	0.875	0.582
	TTF2	0.863			
	TTF3	0.858			
	TTF4	0.803			
Adoption Intention (AI)	AI1	0.796	0.900	0.863	0.644
	AI2	0.805			
	AI3	0.795			
	AI4	0.850			
	AI5	0.763			

Note(s): Items such as “IP5” and “TTF5” were removed due to low loading.

**Table 3:** Discriminant validity (HTMT).

	AI	INN	IP	PCQ	PF	PTR	SE	TTF
AI								
INN	0.413							
IP	0.213	0.134						
PCQ	0.259	0.472	0.084					
PF	0.24	0.056	0.069	0.074				
PTR	0.118	0.071	0.159	0.049	0.117			
SE	0.322	0.056	0.064	0.262	0.14	0.119		
TTF	0.598	0.129	0.139	0.22	0.326	0.328	0.194	

#### 4.2.2. Structural model testing.

For the structural model, multicollinearity between latent variables was first tested. The results showed that the variance inflation factor (VIF) for all paths ranged from 1.000 to 1.035, which is well below the risk threshold of 3.3 [52], indicating that the model does not suffer from serious multicollinearity problems. The predictive ability of the structural model was further validated using PLS. The root mean square error (RMSE) and mean absolute error (MAE) of the model for the main endogenous variable (i.e., AIGC adoption intention) were better than the benchmark linear model, indicating that the model possesses good predictive validity [48].

Path coefficient significance was further tested using 5,000 bootstrap resamples. The results showed that most of the paths significantly held (*p* < 0.05). Among them, PF had a significant positive effect on SE (*β* = 0.151, *p* < 0.05) and TTF (*β* = 0.320, *p* < 0.001), thus supporting paths H1 and H2. perceptions of content quality had a significant positive effect on SE (*β* = 0.265, *p* < 0.001), INN (*β* = 0.438, p < 0.001) and TTF (*β* = 0.216, *p* < 0.001) all had a significant positive effect, thus supporting paths H3-H5; IP had no significant effect on INN (*β* = 0.123, *p* > 0.05) and TTF (*β* = −0.029, *p* > 0.05) and thus did not support paths H6 and H7; PTR had a significant effect on SE (*β* = − 0.141, *p* < 0.05) and TTF (*β* = −0.332, *p* < 0.001) were significantly negative, thus supporting H8 and H9; SE (*β* = 0.202, *p* < 0.001), INN (*β* = 0.323, *p* < 0.001), and TTF (*β* = 0.425, *p* < 0.001) were significantly positively predicted fashion designers’ intention to adopt AIGC, thus supporting paths H10-H12. The relevant path coefficients, t-values and significance levels are detailed in Table 4.

**Table 4:** Structural model.

Hypotheses	PLS paths	Std. beta	T statistics
H1	PF → SE	0.151	2.537^*^
H2	PF → TTF	0.320	6.047^***^
H3	PCQ → SE	0.265	4.760^***^
H4	PCQ → INN	0.438	9.840^***^
H5	PCQ → TTF	0.216	3.929^***^
H6	IP → INN	0.123	1.477^ns^
H7	IP → TTF	−0.029	0.317^ns^
H8	PTR → SE	−0.141	2.497^*^
H9	PTR → TTF	−0.332	6.747^***^
H10	SE → AI	0.202	4.125^***^
H11	INN → AI	0.323	6.798^***^
H12	TTF → AI	0.425	10.454^***^

Note(s): ***p < 0.001; **p < 0.01; ^ns^Not significant.

#### 4.2.3. Analysis of explanatory power and effect sizes.

To evaluate the practical significance of the structural model, the analysis considered not only the significance of path coefficients but also the explanatory power (*R*²) of endogenous variables and the effect sizes (*f*²) of predictors. The results showed that the model explained 38.6% of the variance in Adoption Intention (AI) (*R*² = 0.386), indicating moderate to strong explanatory power. In addition, the R² values for TTF, INN and SE were 0.233, 0.207, and 0.206, respectively, which fall within the acceptable to good range in behavioral research.

The effect size (*f*²) analysis further clarified the actual impact of predictors. For AI, TTF had the largest effect (*f*² = 0.284), which is generally considered a medium effect size [53]. This provides strong support for Hypothesis H12, suggesting that TTF is a key psychological mechanism driving designers’ adoption of AIGC. INN had a small-to-medium effect (*f*² = 0.168), confirming its important influence. By contrast, SE showed a small effect (*f*² = 0.065), indicating statistical significance but limited practical contribution. Among external stimuli, PCQ exerted a significant indirect effect on AI through mediating variables (*f*² = 0.242, medium effect). Perceived Technological Risk (PTR) had small-to-medium negative effects on both TTF (*f*² = 0.141) and SE (*f*² = 0.141), highlighting its suppressive role. Industry Pressure (IP) showed a negligible effect on AI (*f*² = 0.019), consistent with its non-significant path coefficient.

#### 4.2.4. Importance performance mapping analysis (IPMA).

**Fig 2:**
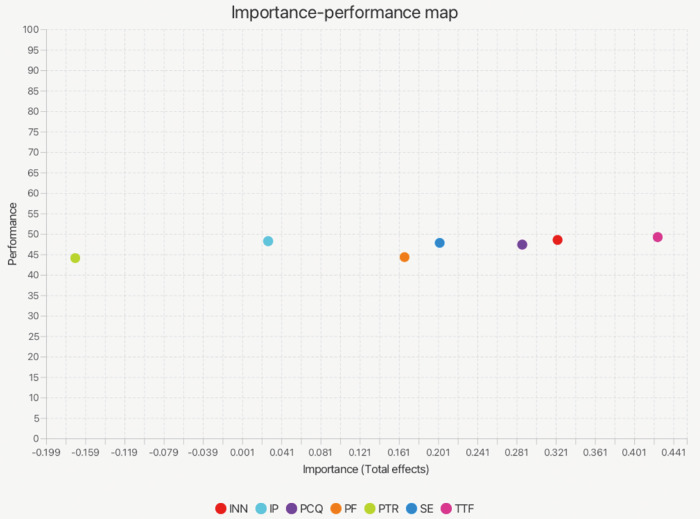
IPMA result.

To further identify key variables influencing fashion designers’ adoption of AIGC, this study used IPMA for follow-up analysis [54]. The methodology integrates the path coefficients (importance) and mean scores (performance) of potential variables to reveal potential constructs that need to be improved despite high impact, thus informing practice optimization. Using AI as the target construct, Fig 2 shows the importance-performance distribution of the predictor variables. The analysis results show that TTF is the most prominent among all variables, ranking first in both Total Effect and Performance Score, indicating that designers generally recognize the usefulness and suitability of AIGC tools in supporting task execution. This indicates that designers generally recognize the usefulness and suitability of the AIGC tool in supporting task execution, and that this perception significantly increases their intention to adopt it. INN ranked second in importance, followed by IP, SE, PCQ, and PF. Although these variables are slightly lower than the TTF in terms of importance dimensions, they also show a high level of influence. Among them, the average performance of PCQ and PF is slightly lower, indicating that designers still have higher expectations for AIGC output quality and personalized support, which suggests that there is still room for optimization of the system in terms of content innovation and user appropriateness. Finally, the PTR has the lowest importance and performance score, indicating that designers’ perception of the potential risks of the AIGC system is relatively weak, and the risk perception has not yet constituted a major resistance to their adoption behavior.

## 5. Discussion

This study integrates the S-O-R model to systematically explore the psychological paths and technology adaptation mechanisms of fashion designers’ adoption of AIGC-aided design. The results show that internal mechanisms (SE, INN, TTF) are key predictors of adoption behavior, while external stimulus variables (e.g., PF, PCQ) indirectly affect adoption intention through the “organismic” path. Among them, TTF was the most prominent in both path coefficients and IPMA importance scores, indicating its centrality in designers’ adoption of AIGC.

TTF not only represents designers’ perception of the matching between system functions and task requirements, but also reflects their evaluation of the effectiveness of technical support in the creative process. When the AIGC system can effectively meet the needs of key design tasks such as idea generation, style control and detail editing, designers are more likely to form a clear perception of the value of the tool and their willingness to adopt it. Especially in the highly task-driven and expression-oriented creative practice of fashion design, the degree of technology suitability has gone beyond the general sense of “ease of use” or “usefulness”, and has become a bridge connecting the tool capability and creative achievements.

This finding not only confirms the TTF theoretical framework proposed by Goodhue and Thompson, but also suggests that the “task responsiveness” of a tool, i.e., its ability to help users achieve stylistic expression, iterative experimentation, and rapid output, has a significant impact on technology adoption behavior in creative work scenarios [30]. When evaluating AIGC tools, designers tend to use “can it help me accomplish my creative tasks” as the main criterion, rather than simply considering the ease of use or innovation of the system’s interface. This mechanism suggests that improving the task fit of the tool is the most direct and effective way to stimulate the technology adoption of the creative community.

At the organism level, SE, INN, and TTF degrees significantly and positively predict designers’ AIGC adoption intentions. Among them, TTF is at the highest level of both importance and performance scores in the IPMA graph, indicating that current designers have generally recognized the utility of AIGC tools in supporting task execution, and that the suitability of technical functions is the key to drive adoption. Fashion design is a complex cognitive activity that combines technical manipulation and emotional expression, and designers are more inclined to use AIGC-assisted design if they know that they are capable of using AI tools and believe that they are better able to reinforce creative inspiration and accomplish specific design tasks. This finding corroborates Bandura’s “efficacy belief-behavioral tendency” pathway [28], but also reflects the fact that innovative individuals are more inclined to exhibit exploratory behaviors when faced with technological uncertainty [55].

The significant negative effect of perceived technological risk suggests that fashion designers are particularly concerned about the uncontrollability of generated content, stylistic deviations, and potential copyright conflicts when using the AIGC tool. These concerns undermined their perceived efficacy and adoption confidence in the system. In particular, the study found that IP had no significant effect on INN or TTF, hence hypotheses H6 and H7 were not substantiated. This finding may reflect the distinct adoption behavior of fashion designers as a creative group. Unlike traditional technology adoption models, which frequently see external pressure as a key driver, fashion designers, who rely heavily on intrinsic motivation and original expression, may not translate competitive industry pressure into positive evaluations of new technologies or increased creative impulses. Instead, such pressure may be interpreted as a background component rather than a direct psychological stimulation. Their adoption decisions appear to be influenced more by whether the tool meets core creative task needs (TTF) and confidence in their own abilities (SE) than by external trends. This finding is consistent with previous studies on knowledge workers and creative professions, indicating that their behavior is more strongly influenced by intrinsic motivation and perceived value of tools, while being less sensitive to broad industry pressure.

The study finds that PCQ and PF score high in importance but lower in performance in the IPMA analysis. This gap carries strong practical implications, as it highlights the main tension between current AIGC tools and fashion designers’ expectations. Designers recognize that personalization and high-quality content are central to creative work, yet their actual experience with existing tools falls short of these standards. Several reasons may explain this gap.1. Current general-purpose AIGC models (e.g., Midjourney) are not developed specifically for fashion design. As a result, they struggle to capture professional needs such as fabric texture, garment structure, and craftsmanship details, leading to weaker content quality. 2. Existing personalization features rely mostly on surface-level prompt adjustments, making it difficult for the system to learn and adapt to designers’ unique style systems, color preferences, and design logic. 3. Designers have a strong need for control in the creative process, but the inherent unpredictability of AIGC outputs reduces their sense of control and satisfaction regarding PCQ and PF. Therefore, this performance gap does not imply that PF and PCQ are unimportant. On the contrary, it identifies them as the key directions for future improvement. Developers should focus on training domain-specific models, introducing more fine-grained style control parameters, and enhancing user intervention in the generation process to improve both personalization and professional content quality.

### 5.1. Theoretical implications

This study makes the following contributions at the theoretical level:

1. This study introduces the S-O-R framework into research on AIGC adoption and demonstrates its applicability and explanatory power in the creative design context. By uncovering the unique adoption logic of AIGC as an emerging creative technology, the study highlights the importance of considering both technology type and task nature in adoption research. This contributes to the refinement and contextualization of the S-O-R framework. Another key theoretical contribution lies in the contextual application of established theories. Instead of mechanically applying abstract constructs from classic models, the study integrates core ideas from SDT and DOI into the context of AIGC-assisted fashion design. Specifically, the autonomy need from SDT is operationalized as measurable PF, and the competence need as PCQ. Likewise, DOI’s compatibility attribute is captured through TTF, a construct widely validated in information systems research.
2. This study integrates a number of psychological and technical variables that have been less frequently addressed in previous studies, including PF, PCQ, SE, INN, TTF, and PTR. Unlike traditional information systems research, which tends to focus on system reliability and ease of use, this study highlights PF and PCQ as key stimulus variables. These constructs capture the essence of AIGC as both a content-generation tool and a medium for creative expression, standing in sharp contrast to contexts that emphasize information processing or transaction completion. For example, in e-commerce studies using the S-O-R framework, personalization usually refers to the accuracy of product recommendations [56]. In this study, however, personalization is directly tied to designers’ sense of stylistic identity and creative autonomy, giving it a deeper psychological meaning.
3. This study emphasizes the central role of TTF and individual innovativeness in driving AIGC adoption. The findings confirm that TTF is the strongest psychological mechanism driving AIGC adoption. This is consistent with previous research on task-oriented technology such as enterprise systems (ERP) and online learning platforms [10,57]. With contrast, with more general-purpose technologies aimed at entertainment or social engagement (such as social media), TTF is frequently secondary to perceived usefulness [58]. TTF has a greater impact on professional fashion design. This implies that for systems built to support complicated, unstructured professional tasks like AIGC, alignment with basic creative workflows becomes the foundation of user decisions and may even outweigh perceived utility. Furthermore, the study concludes that IP has no substantial effect. This is in contrast to several research on organizationally mandated technologies [36]. The distinction demonstrates that in domains reliant on individual innovation and aesthetic judgment, external normative pressure has little psychological impact on technology evaluation. Instead, adoption decisions are mostly influenced by the extent to which technologies suit intrinsic creative requirements and individual innovativeness. This demonstrates the theoretical restriction of seeing creative professionals as equals to traditional technology consumers.
4. In contrast to previous studies in which perceived risk has primarily served as an extrinsic inhibitor [39], the present study further found that perceptions of technological risk not only weakened adoption intentions directly, but also had an indirect effect through pathways such as lowering self-efficacy and weakening task fit. This suggests that designers will be more cautious in competence judgment and trust building when there is uncertainty in AIGC output, thus providing a more nuanced theoretical addition to the study of technology adoption mechanisms from a risk perspective.

### 5.2. Practical implications

This study provides specific practical insights for the popularization and application of AIGC technology in the fashion design industry, especially for AIGC tool developers and platform operators with important reference value.

1. Improving task fit and innovativeness support is the key to stimulate designers’ adoption intentions. This study found that TTF was outstanding in both importance and performance dimensions, indicating that the current AIGC tool has been widely recognized by designers in terms of task execution efficiency and functional alignment. Especially in terms of integration with design process software such as CLO3D and Photoshop, the platform should further strengthen the responsiveness of the AIGC tool to specific task requirements and improve the task matching of modules such as intelligent generation, sketch recognition, and color matching, so as to promote a synergistic enhancement of innovativeness and tool utility.
2. PF and PCQ significantly affect designers’ psychological identification with AIGC. Although the performance scores of these two variables in the IPMA graph are relatively low, indicating that there is still room for improvement in the levels of “personalized output” and “content credibility”. To enhance PF, AIGC tool developers can move beyond basic prompt-based generation and introduce more refined user control and learning mechanisms. For example, a personal style library could be created, allowing designers to upload their past work so that the AI can learn their unique color palettes, silhouette preferences, and pattern styles, enabling truly personalized outputs. Instead of a single text box, an advanced prompt control panel could be provided with sliders tailored to fashion design, such as creativity level, stylistic intensity, and fabric texture, giving designers greater control over the results. In addition, a feedback loop could be built in so that after each output, designers are offered refinement options. This would allow the system to adjust in real time based on feedback, gradually producing more precise and personalized results.
3. The study found that technology risk perception has a significant inhibitory effect on adoption intention, which reminds platform operators to proactively reduce users’ concerns about potential risks such as uncontrollable output results, infringement of originality, and content distortion when promoting AIGC tools. Specifically, the credibility and security of the system can be enhanced by improving the interpretability of the generated content, providing options for result correction, and setting up style protection and copyright alert functions. For example, setting up style protection and copyright alert functions, such as integrating copyright-checking and source-tracing tools that detect similarity between generated outputs and existing well-known designs, and sending alerts to designers to avoid potential disputes.
4. This study also has substantial implications for educational reform in design schools. As SE and INN are identified as significant psychological drivers of AIGC adoption, design education should shift away from traditional skill training and emphasize on developing students’ technological confidence and innovative thinking when working with intelligent tools. AIGC can be used as a typical assistance tool in core courses including design principles and creative concept creation. Project-based learning allows students to obtain hands-on experience, which boosts their self-efficacy in utilizing AIGC. Furthermore, advanced AI courses can inspire students to critically analyze the role of AI in the creative process, motivating them to investigate how human design and AI creation might complement one another rather than considering AI as a simple substitute.

### 5.3. Limitations and suggestions for future research

Although this study has achieved some results in both theory and practice, there are still some limitations, and future research can be further expanded and improved in the following aspects.

1. The sample of this study is limited to fashion designers in mainland China, which can reflect the current industry trends to a certain extent, but has geographical limitations in terms of culture, policy and technology use environment. In the future, we can introduce samples of designers from other countries or regions (e.g., Japan, South Korea, Europe, etc.) to carry out cross-cultural comparative studies, and explore how cultural differences affect the adoption path and cognitive mechanisms of AIGC, so as to enhance the external applicability of the results of the study.
2. Although this study has included the variable of IP to capture certain external social influences, it mainly reflects macro-level industry trends, and its consideration of internal factors (e.g., teamwork, leadership support, and innovation culture) and social context variables (e.g., customer interactions, and technological norms) is still relatively limited. In view of the fact that fashion design work is highly dependent on design teamwork and customer feedback, future research can further introduce multilevel organizational factors and social cognitive mechanisms, such as organizational norms, leadership styles, and task coordination efficiencies, in order to enhance the explanatory power of the model for complex practice environments.
3. The cross-sectional questionnaires used in this study reveal the structural relationship between variables, but it is difficult to capture the evolutionary path of designers’ adoption behavior during the use of AIGC tools. It is suggested that future research can combine the situational experimental method, behavioral recording method, or tracking survey, for example, by setting up an A/B testing environment or video-recording behavioral analysis, in order to explore behavioral fluctuations and changes in psychological mechanisms during the actual use of the AIGC tool.
4. This study focuses primarily on the overall psychological mechanisms that influence adoption intention, without considering the potential moderating effects of demographic characteristics (e.g., gender, age) or work background variables (e.g., corporate designers vs. independent designers). The suggestion to conduct a multigroup analysis is indeed valuable. However, due to the small sample size, accurate group comparisons may lack statistical power. Future studies with bigger samples could systematically explore these moderating effects, showing both the generalizability and subgroup variations in the AIGC adoption model. Such findings would provide a solid foundation for more targeted intervention techniques.

## Supporting information

S1 Table Measurement items and sources. (DOCX)

S1 Data Data. (XLSX)
