# Supplementary material for: Analyzing the adoption of AIGC tools in fashion design: An S-O-R framework integrating task–technology fit
Source: PLoS One. 2025 Oct 27;20(10):e0335522. doi: 10.1371/journal.pone.0335522 (PMC12558467; doi:10.1371/journal.pone.0335522)
Supplement: S1 Table — (DOCX) [file pone.0335522.s001.docx]

S1 Table. Measurement items and sources

|  | Items | Source (Reference/Adapted) |
| --- | --- | --- |
| Personalized Fit (PF) | PF1: I believe the AIGC system has the potential to offer suggestions based on my design style preferences. | [1,2] |
|  | PF2: The AIGC system may have a certain degree of adaptability to align with my design habits over time. |  |
|  | PF3: Although current outputs are still general, I expect AIGC content to become more tailored to my individual design needs. |  |
|  | PF4: When interacting with AIGC, I sometimes sense that the system shows early signs of understanding my design preferences. |  |
|  | PF5: I believe the AIGC system has the potential to better align with my personalized design preferences in the future. |  |
| Perceived Content Quality (PCQ) | PCQ1: I believe the content generated by the AIGC system is accurate. | [3] |
|  | PCQ2: The content provided by the AIGC system is complete and contains all the information I need. |  |
|  | PCQ3: The content generated by the AIGC system is clear and easy to understand, and the presentation is complete. |  |
|  | PCQ4: The content provided by the AIGC system is innovative and design inspiring. |  |
|  | PCQ5: I am generally satisfied with the quality of the content generated by the AIGC system. |  |
| Industry Pressure (IP) | IP1: I feel the pressure of technological competition from my industry peers in the use of AIGC tools. | [4,5] |
|  | IP2: Influences from design circles or professional organizations prompted me to consider using the AIGC system |  |
|  | IP3: I am concerned that if I don't adopt the AIGC tool, I will lose my competitive edge in the industry. |  |
|  | IP4: More and more design brands in the market are starting to adopt AIGC, which makes me feel compelled to follow suit. |  |
|  | IP5: My clients/supervisors expect me to use AIGC tools to improve design efficiency or quality. |  |
| Perceived Technological Risk (PTR) | PTR1: I am concerned that the content generated by the AIGC system may contain errors or inaccuracies. | [6] |
|  | PTR2: I am concerned that using AIGC may result in my design style not being adequately represented. |  |
|  | PTR3: I believe that using AIGC increases the risk of a lack of control over the results. |  |
|  | PTR4: I am concerned that the content generated by AIGC may infringe the intellectual property rights or originality of others. |  |
|  | PTR5: I feel that AIGC systems exhibit unpredictability in complex design tasks. |  |
| Self-Efficacy (SE) | SE1: I believe I can skillfully use AIGC tools to assist in design tasks. | [7,8] |
|  | SE2: I am able to complete creative tasks using AIGC independently even without help. |  |
|  | SE3: I am confident that I can find solutions to design problems through AIGC technology when I face difficulties. |  |
|  | SE4: I feel that I can control the rhythm of using AIGC tools and integrate them into my daily design process. |  |
|  | SE5: I am capable of evaluating and selecting AIGC tools and features that suit my creative needs. |  |
| Innovativeness (INN) | INN1: I enjoy trying to incorporate emerging technologies such as AIGC into my design work. | [9,10] |
|  | INN2: I am more likely to be the first to try new design tools or techniques that are not yet widely available than others. |  |
|  | INN3: I enjoy exploring challenging or unprecedented design expressions. |  |
|  | INN4: I often generate unique design ideas and am willing to put them into practice. |  |
|  | INN5: I believe that using AIGC technology for creative expression is a potential design breakthrough. |  |
| Task-Technology Fit (TTF) | TTF1: The AIGC system meets my mission-critical needs in fashion design. | [11,12] |
|  | TTF2: The functionality of the AIGC tools fits well with my design process. |  |
|  | TTF3: Using AIGC helps me to complete complex design tasks more efficiently. |  |
|  | TTF4: The AIGC system can effectively support my daily creative tasks (e.g. image generation, style matching, etc.). |  |
|  | TFF5: I think the functions of AIGC are suitable for solving the various problems I encounter in the design process. |  |
| Adoption Intention (AI) | AI1: I would like to use the AIGC system in my design work if conditions permit. | [13,14] |
|  | AI2: I plan to use AIGC as a support tool in my future design projects. |  |
|  | AI3: I think I will continue to use AIGC for my daily fashion design tasks. |  |
|  | AI4: AIGC is a new technology that I would like to actively try in my future creative work. |  |
|  | AI5: I have a strong desire to incorporate AIGC into my design practice. |  |

**Reference**

1. Komiak, Benbasat. The Effects of Personalization and Familiarity on Trust and Adoption of Recommendation Agents. MIS Quarterly. 2006;30: 941. doi:10.2307/25148760

2. Tam KY, Ho SY. Understanding the Impact of Web Personalization on User Information Processing and Decision Outcomes. MIS Quarterly. 2006;30: 865–890. doi:10.2307/25148757

3. Wixom BH, Todd PA. A Theoretical Integration of User Satisfaction and Technology Acceptance. Information Systems Research. 2005;16: 85–102. doi:10.1287/isre.1050.0042

4. Teo HH, Wei KK, Benbasat I. Predicting Intention to Adopt Interorganizational Linkages: An Institutional Perspective. MIS Quarterly. 2003;27: 19–49. doi:10.2307/30036518

5. Oliveira T, Thomas M, Espadanal M. Assessing the determinants of cloud computing adoption: An analysis of the manufacturing and services sectors. Information & Management. 2014;51: 497–510. doi:10.1016/j.im.2014.03.006

6. Xiao B, Benbasat I. E-Commerce Product Recommendation Agents: Use, Characteristics, and Impact. MIS Quarterly. 2007;31: 137–209. doi:10.2307/25148784

7. Compeau DR, Higgins CA. Computer Self-Efficacy: Development of a Measure and Initial Test. MIS Quarterly. 1995;19: 189–211. doi:10.2307/249688

8. Marakas GM, Yi MY, Johnson RD. The Multilevel and Multifaceted Character of Computer Self-Efficacy: Toward Clarification of the Construct and an Integrative Framework for Research. Information Systems Research. 1998;9: 126–163. doi:10.1287/isre.9.2.126

9. Hurt HT, Joseph K, Cook CD. SCALES FOR THE MEASUREMENT OF INNOVATIVENESS. Human Comm Res. 1977;4: 58–65. doi:10.1111/j.1468-2958.1977.tb00597.x

10. Zhou J, George JM. WHEN JOB DISSATISFACTION LEADS TO CREATIVITY: ENCOURAGING THE EXPRESSION OF VOICE. Academy of Management Journal. 2001;44: 682–696. doi:10.2307/3069410

11. Gebauer J, Ginsburg M. Exploring the Black Box of Task−Technology Fit: The Case of Mobile Information Systems.

12. Goodhue DL, Thompson RL. Task-Technology Fit and Individual Performance. MIS Quarterly. 1995;19: 213–236. doi:10.2307/249689

13. Venkatesh V, Morris MG, Davis GB, Davis FD. User Acceptance of Information Technology: Toward a Unified View. MIS Quarterly. 2003;27: 425. doi:10.2307/30036540

14. Venkatesh V, James Y. L. Thong, Xin Xu. Consumer Acceptance and Use of Information Technology: Extending the Unified Theory of Acceptance and Use of Technology. MIS Quarterly. 2012;36: 157. doi:10.2307/41410412
